# Effects of Altitude on Chronic Obstructive Pulmonary Disease Patients: Risks and Care

**DOI:** 10.3390/life11080798

**Published:** 2021-08-07

**Authors:** Thomas Georges, Camille Le Blanc, Sophie Ferreol, Pierre Menu, Marc Dauty, Alban Fouasson-Chailloux

**Affiliations:** 1 CHU Nantes, Service de Médecine Physique et Réadapatation Locomotrice et Respiratoire, 44093 Nantes, France; thomas.georges@chu-nantes.fr (T.G.); camille.leblanc@chu-nantes.fr (C.L.B.); sophie.ferreol@chu-nantes.fr (S.F.); pierre.menu@chu-nantes.fr (P.M.); marc.dauty@chu-nantes.fr (M.D.); 2 CHU Nantes, Service de Médecine du Sport, 44093 Nantes, France; 3 IRMS, Institut Régional de Médecine du Sport, 44093 Nantes, France; 4 Inserm, UMR 1229, RMeS, Regenerative Medicine and Skeleton, Université de Nantes, ONIRIS, 44042 Nantes, France

**Keywords:** chronic obstructive pulmonary disease, hypoxemia, altitude, air travel

## Abstract

Air travel and altitude stays have become increasingly frequent within the overall population but also in patients suffering from chronic obstructive pulmonary disease (COPD), which is the most common respiratory disease worldwide. While altitude is well tolerated by most individuals, COPD patients are exposed to some serious complications, that could be life-threatening. COPD patients present not only a respiratory illness but also frequent comorbidities. Beyond oxygen desaturation, it also affects respiratory mechanics, and those patients are at high risk to decompensate a cardiac condition, pulmonary hypertension, or a sleep disorder. Recently, there has been considerable progress in the management of this disease. Nocturnal oxygen therapy, inhaled medications, corticosteroids, inspiratory muscle training, and pulmonary rehabilitation are practical tools that must be developed in the comprehensive care of those patients so as to enable them to afford altitude stays.

## 1. Introduction

More and more people are traveling at high altitude. According to the International Civil Aviation Organization (ICAO), 4.5 billion passengers traveled by air in 2019 [1]. Democratization of air transports, improvement of reception conditions, and development of mountain tourism expose travelers to altitude and to its health consequence, which can be potentially serious.

Chronic obstructive pulmonary disease (COPD) is the most common chronic respiratory disease worldwide. It is a very common disease characterized by persistent respiratory symptoms, associated to airflow limitation assessed by spirometry (defined by the presence of a post-bronchodilator Forced Expiratory Volume in 1 s (FEV1)/Forced Vital Capacity (FVC) < 0.7) [2]. Due to the aging of the population and multiple toxic exposures (tobacco smoking, occupational exposure, air pollution), its prevalence is increasing throughout the world and has been recently evaluated at 12% [3]. Although this disease decreases health status and causes significant disabilities [4], improving cares permit to reduce symptoms and to improve exercise tolerance and quality of life. Thus, those patients are also concerned by altitude travel. This is a frequent question in clinical practice to evaluate the possibility or not for those people to go on a journey at moderate altitude. Indeed, it has been described that altitude reduces exercise endurance in COPD people [5,6]. Ergan et al. performed a study, pointing that, even among pulmonologists, a standard approach is lacking a “fit to fly” concept [7]. Due to a particular pathophysiology and comorbidities, such as cardiovascular disease, musculoskeletal impairment, or diabetes mellitus, altitude exposes COPD patients to an increased risk of respiratory or cardiac decompensation.

In this article, we performed a narrative review to describe assessment of those patients before traveling at altitude and ways to improve their care.

## 2. Literature Search

We searched articles in the medical databases PubMed, ScienceDirect, and Google Scholar in May 2021. Multiple searches were carried out using the following MeSH (“COPD”) AND (“Altitude” OR “Air travel”). We assessed titles or abstracts of underlined articles, and then the full text of potentially relevant articles were retrieved. After identification of key articles, their references were also studied for further information sources. The search was performed independently by 2 assessors (TG, AFC) to judge titles and abstracts of potentially relevant articles, and then the full-text articles were retrieved. All relevant articles were read independently in full text by the two researchers (TG, AFC).

## 3. Pathophysiology of Altitude

When climbing in altitude, decrease of inspired oxygen tension (PiO_2_) leads to a fall in alveolar oxygen tension (PAO_2_) and, thus, a decrease in arterial oxygen tension (PaO_2_) [8]. The changes in PiO_2_ due to changes in altitude can be determined by the equation:

PiO_2_ = FiO_2_ × (Patm − PH_2_O), (1)

where FiO_2_ is the fraction of inspired oxygen (0.21 in atmospheric air), Patm is the atmospheric pressure (760 mmHg at sea level), and PH_2_O is the partial pressure of water (47 mmHg at 37 °C). The decline of PaO_2_ is dependent on the ventilatory response to hypoxia [8].

This decrease in PaO_2_ leads to adaptation mechanisms to avoid a potentially dangerous hypoxemia. Hypoxemia is detected by chemoreceptors located in the carotids’ wall. Then, the signal is transmitted to the brainstem where the nerve cells responsible for the ventilation control are located [8].

**Figure 1:**
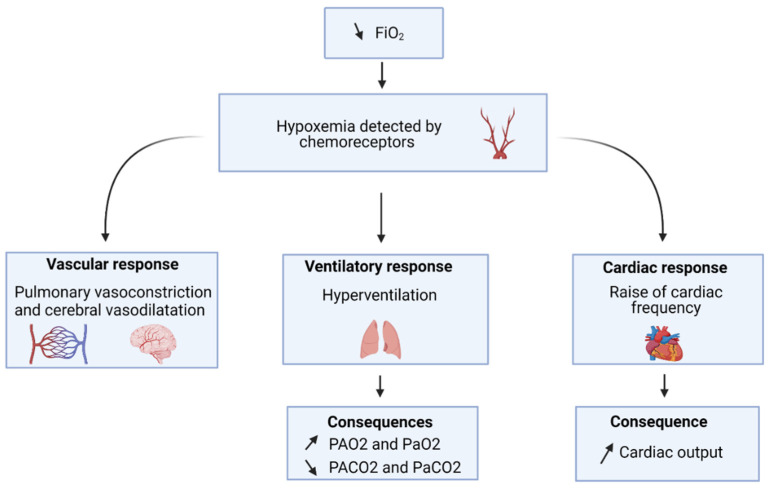
Cardio-circulatory and respiratory systems initial response to hypoxia.

There are 2 phases in the compensatory response, according to short-term exposure and long-term altitude exposure. The initial response to hypoxia is to raise minute ventilation, avoiding as much as possible the fall of PAO_2_ and, thus, PaO_2_ (Figure 1). This leads to a decrease in PaCO_2_ and then respiratory alkalosis [8]. Cardiac response consists of the raise of cardiac frequency and stroke volume, allowing an increase of cardiac output [9]. An increase in diuresis serves to facilitate bicarbonate losses and, then, to stimulate increased breathing. The pulmonary arteries constrict in response to tissue hypoxia, to redirect blood flow to the alveoli containing the highest oxygen content [8]. Finally, there is a shift of the oxygen-hemoglobin dissociation curve to the left, allowing oxygen to bound on hemoglobin but limiting delivery of oxygen to the tissues [10]. Then, when exposure to hypoxia is prolonged, other compensatory mechanisms develop, and acclimatization begins. Indeed, erythropoiesis allows the increase in the amount of red blood cells and, therefore, improves the transport of oxygen [8]. Several growth factors upregulate the capillary bed by inducing angiogenesis and promoting oxidative metabolism [11]. There is also an increase in 2,3-diphosphoglycerate, allowing the return of oxygen-hemoglobin dissociation curve at its initial level, thus allowing the distribution of the oxygen to the tissues [10].

## 4. Altitude-Hypoxemia Assessment in COPD Patients

Healthy individuals have a higher baseline PaO_2_ than COPD patients. The change in PaO_2_ in healthy people interests the flat portion of the oxyhemoglobin curve, so the decrease in oxygen saturation (SpO_2_) will be moderate. Conversely, in the subjects with a reduced baseline PaO_2_, such as in COPD, the decreased of PaO_2_ will concern the steeper part of the oxyhemoglobin curve; thus, the decrease in oxygen saturation could be much more severe [12]. There are several tools to help identify which patients will need oxygen in such conditions. Most of the performed studies concerned the in-flight need of oxygen, and there is almost exclusively data for such altitudes. Indeed, cabin pressurization during a flight allows an altitude equivalent of about 2438 m (8000 ft) [13,14] and, thus, an exposure to a FiO_2_ of about 15.1%.

### 4.1. Pulse Oximetry

The easiest way to assess respiratory status in COPD patients is pulse oximetry. Yet, Akero et al. concluded that SpO_2_ measurement at rest at sea level could not discriminate COPD patients will need oxygen during flight, due to a lack of sensitivity [15]. Indeed, by dividing COPD patients into 4 groups according to SpO_2_ (>95%, 92–95% without additional factors, 92–95% with cardiac disease or FEV1 < 50% predicted or lung cancer or cerebrovascular disease, and <92%), the need of in-flight oxygen was, respectively, 30%, 67%, 70%, and 83%. Robson et al. also showed that 28% of COPD patients having SpO_2_ between 92 and 95% and no additional risk factors (as described above) desaturated during hypoxic challenge testing [16]. However, Ling et al. found good agreement between rest SpO_2_ and SpO_2_ during hypoxic challenge testing [17].

### 4.2. Blood Gases

Christensen et al. evaluated the contribution of blood gases in the assessment of hypoxemia in altitude in COPD patients. In a group of 15 subjects with a FEV1 < 50% with a baseline PaO_2_ > 9.3 kPa (70 mmHg), they found that 33% and 66% of patients desaturated below 6.7 kPa (50 mmHg) at 2438 m and 3048 m, respectively. There was no correlation between PaO_2_, FEV1, and Carbon monoxide transfer capacity (TLCO) at sea level and PaO_2_ at 2348 m [18]. Akero also demonstrated that a pre-flight PaO_2_ > 9.3 kPa (70 mmHg) did not predict secure in-flight PaO_2_ [19].

### 4.3. Pulmonary Function Testing

Several studies assessed the contribution of pulmonary function testing to predict hypoxemia in COPD patients [16,17,18,19,20,21]. Most of the studies assessing spirometry failed to predict hypoxemia [16,17,18]. In those studies, FEV1 and FVC did not correlate with the fall of PaO_2_ at altitude.

Results concerning TLCO are conflicting, even if this test seems interesting. In the study by Kelly et al., Pearson’s correlation was calculated at 0.74 between TLCO and resting SaO_2_ at altitude [20]. Dellweg et al. found good agreement between diffusion capacity and nadir SaO_2_ in COPD patients (mean FEV1 41%) [21]. However, Christensen et al. did not bring out a correlation between diffusion capacity and PaO_2_ at 2348 m of altitude [18]. In the study by Ling et al., there was a weak correlation between diffusion capacity and SpO_2_ during hypoxic challenge testing [17].

### 4.4. 6-min Walking Test

The 6-min walk test (6MWT) is often used for the evaluation of exercise capacity in COPD patients [22]. BODE score, commonly used to assess prognosis in COPD, integrates this parameter [23]. It is a simple and practical field test, it can be rapidly realized, and it does not require sophisticated equipment. Walking tests show interesting results for in-flight assessment. Chetta et al. highlighted correlation between desaturation induced by 6MWT and hypoxic challenge testing (r = 0.52, *p* < 0.01) [24]. Edvardsen et al. constructed an algorithm which had a sensitivity of 100% and specificity of 80% for recommending a supplementation in oxygen during a flight [25]. In this study, COPD patients who had a baseline SpO_2_ > 95% and SpO_2_ after 6MWT > 84% were able to travel without further assessment. In-flight oxygen therapy was recommended if baseline SpO_2_ was less than 92% or if SpO_2_ during 6MWT was less than 84%. It should be noted that patients with a baseline SpO_2_ > 95% accounted for 12% of their population.

### 4.5. Equations

There are several equations to determine in-flight PaO_2_, taking into account FEV1, FEV1/FCV, TLCO, and PaCO_2_. It is an attractive option due to its simplicity. In 1993, a meta-analysis found good agreement between estimation and PaO_2_ at 8000 ft in COPD patients [26]. More recently, Bradi et al. performed a new meta-analysis and showed poor agreement between equations and PaO_2_ measurement during a Hypoxic Challenge test (HCT) [27]. Those equations usually overestimate altitude induced-hypoxemia and can then lead to excessive prescription of oxygen [28].

### 4.6. Hypoxic Challenge Testing

This test currently constitutes the benchmark. Described by Gong et al. in 1984, it consists in inhaling air with an FiO_2_ of 15% (and 85% nitrogen) [29]. This can be obtained thanks to different devices allowing the modification of the composition of inspired air, such as a pre-mixes cylinder provided from medical gas providers, a hypoxic gas generator connected to a face mask, or closed chamber or a 40% venturi oxygen mask used with pure nitrogen (resulting in a gas mixture with about 15% of oxygen). The test lasts 20 min, the duration after which the oxygen equilibrium is usually reached. It has the advantage to be simpler in practice than using a hypobaric chamber, and the results seem comparable [30]. There is a good correlation between SpO_2_ measured in HCT and measured in-flight [31]. British Thoracic Society [32] and French Society of Pneumology [33] have made some proposals about HCT for the assessment of “fitness to fly”. Lately, Ergan et al. have proposed a practical approach advising to perform this test if baseline PaO_2_ was less than 70 mmHg (10,5 kPa) (or SaO_2_ < 95%), if there is desaturation under 84% at 6-min walk test, or if there are some risk factors, such as FEV1 < 1.5 L or 30% predicted, bullous lung disease, comorbid conditions, that may worsen with hypoxemia, and significant symptoms during a previous air travel [34]. The main drawback of HCT is that it must be conducted in a specialized center with advanced equipment.

## 5. Others Causes of Dyspnea Induced by Altitude in COPD Patients

COPD patients have an increased risk of in-flight dyspnea [35]. Some recent studies have put forward some evidence to hypothesize that in-flight dyspnea is not related to hypoxemia in COPD patients. In a study by Edvardsen et al. in 2013, PaO_2_ during HCT was not correlated to in-flight symptoms [36]. More recently, Dellweg et al. did not find association between hypoxemia during a hypobaric flight simulation and perception of dyspnea [21]. Indeed, mechanisms of dyspnea are, therefore, complex and need a comprehensive assessment.

### 5.1. Respiratory Mechanism

Beyond the desaturation and hypoxemia induced by the decrease of alveolar PO_2_, a consequence of hypobaric hypoxemia is hyperventilation, as previously mentioned. Moreover, while pressure decreases, the gas volume increases, according to Boyle law, and then is likely to reduce vital capacity and to increase residual volume [37]. Those two phenomena could induce dynamic distention, which itself leads to increase work of breathing and exhaustion of respiratory muscles [38]. Furthermore, dynamic distension induces decrease of inspiratory capacity, which has been related to dyspnea and decrease of exercise capacity [39].

Altitude effects on bronchoconstriction are unequivocal. Indeed, while the lower density of inspired air could improve airflow dynamic, inhalation of cold air may worsen bronchoconstriction by mucus dehydration [40].

Another risk, against which patients must be warned, is the pneumothorax induced by altitude. Expansion of air in a bulla (due to Boyle law) can cause tissue tear and, thus, worsen hypoxemia [41]. However, pressure change with ascent is relatively slow, and then the probability of pneumothoraces seems moderate [40].

### 5.2. Cardio-Vascular Adaptation

Hypoxic vasoconstriction results in a rise of right ventricular afterload, which might lead to right ventricular dysfunction [42]. COPD patients are at greater risk to develop pulmonary hypertension (PH), even at low altitude. Prevalence of pulmonary hypertension depends on COPD stage and PH definition, but it could represent up to half of patients with advanced disease [43]. Lichtblau et al. have observed an increase in trans-tricuspid pressure gradient (from 23 to 32 mm Hg) and a decrease in right ventricle charge area change (from 45 to 38%) in a cohort of COPD patient stage II–III (mean FEV1 57%) after one night at 2590 m of altitude [44]. The additional load on right ventricle probably increases the risk to develop symptomatic pulmonary hypertension in those predisposed patients.

The rise of pressure in pulmonary vascular system could also worsen right-to-left shunt and then might impair gas exchange efficacy. Prevalence of right-to-left shunt reached 75% at 3100 m in a group of 87 COPD patients [45].

Lastly, sympathetic activation is responsible for increasing heart rate, blood pressure, and cardiac output [46] and may exacerbate coexisting medical conditions, such as coronary heart disease or atrial fibrillation. There is frequently a comorbid heart disease associated with COPD. Up to 15% of COPD could have heart failure, and 39% a coronary disease [47]. Even if altitude seems safe for patients with stable coronary heart disease [48,49], it could result in an earlier onset of angina symptoms due to increased myocardial oxygen consumption [50]. Furthermore, altitude might aggravate arrhythmias, due to sympathetic stimulation, especially during exercise [51]. Regarding the prevalence of atrial fibrillation in COPD patients (14% according to Roversi et al.) [47], they should be warned about this risk. So far, literature identified mainly supraventricular and ventricular extrasystoles [15,29]. In a recent study, 1 out of 24 COPD patients had three runs of ventricular tachycardia during a night at 2048 m [52]. Nevertheless, another recent study in COPD patients traveling at 3100 m did not reveal an increase incidence of signs of ischemia nor arrhythmia during exercise, although statistically significant ST-depressions below the threshold of clinical relevance were detected [53].

## 6. Other Considerations

### 6.1. Sleep

Even in healthy individuals, altitude might induce periodic breathing, sleep disturbances, and nocturnal hypoxemia. Slow-wave sleep is reduced, number of arousals is greater, and periodic ventilation of Cheynes-Stokes may occur. Rapid Eyes Movements stage remains the same [54,55].

In 2019, Lashtang et al. studied effects of altitude on sleep in COPD patients at 1650 and 2590 m [56]. Polysomnography revealed a reduced SpO2 (medians 89% and 85% versus 92% at 490 m), a higher apnea-hypopnea index (respectively, 26.8 et 55.7/h versus 15.4/h), mostly due to central events, and, at 2590 m, sleep efficiency and slow-wave sleep were reduced (respectively, 59% and 17% of total sleep time versus 72% and 20%).

These findings are consistent with the ventilation instability due to hypoxic stimulation of ventilation and decrease in CO_2_ reserve. Authors hypothesized that the greater propensity to central sleep apnea reflects an enhanced neural respiratory drive at higher altitudes in COPD patients. This could occur in addition to mild heart failure, common in those patients, which may have resulted in a prolonged circulatory time. Indeed, circulation disturbances participate in genesis of central sleep events [57].

Recently, Tan et al. studied the effects of oxygen therapy during sleep in individuals who had moderate COPD (Global Initiative for Obstructive Lung Disease stage II or III) [58]. In their randomized placebo-controlled crossover trial, administration of nocturnal oxygen therapy (3 L/min) resulted in decreased hypoxemia and decreased apnea-hypopnea index, as well as improved subjective sleep quality.

### 6.2. Venous Thromboembolism

An increase in thromboembolic events has been described in a cohort of soldiers stationed in altitude, with a relative risk of 44 for deep vein thrombosis and 65.9 for pulmonary embolism [59]. A pro-thrombotic state induced by altitude is suspected but not confirmed [60]. However, most of those thrombosis occurred at an altitude of more than 5700 m. Reduced blood plasma volume and rise in hematocrit (due to dehydration and erythropoietin release) probably contributed to this phenomenon [61,62].

Air travel is believed to be a risk factor for pulmonary embolism. Lapostolle et al. studied pulmonary embolism cases in passengers arrived at Charles de Gaulle Airport, France, and found an increase risk with a greater distance traveled [63]. Similar studies exist for Sidney’s and Madrid’s airports [64]. Schwartz et al. performed 2 studies which found a relative risk of 2.2 (95%CI 1.3–5.3) to develop a thromboembolic disease after an air travel [65,66]. This risk is likely to continue 2 or 3 weeks after the end of the travel [67,68]. Hypoxia, venous stasis, and dehydration probably play a role at different levels. To our knowledge, there is no specific data about COPD patients.

## 7. Management of COPD Patient Wishing to Travel in Moderate-Altitude

Firstly, it seems crucial to refer COPD patients to a respiratory physician before traveling. BTS recommends to screen, during a specific medical assessment, patients with a severe (FEV1 < 50%) or a poorly controlled obstructive pulmonary disease with exacerbations, a bullous disease, a restrictive lung or chest wall conditions, a pulmonary hypertension, or a comorbid condition which might worsen by hypoxemia (cerebrovascular or cardiac disease). Patients already requiring long term oxygen treatment, having an active cancer with lung involvement, or having recently received hospital treatment for a respiratory condition should also be carefully assessed [32]. Medications, before exposition to altitude and possible induced symptoms, should also be evaluated. Because of a risk to increase pulmonary vascular resistance and due to frequent cardiovascular comorbidities, patients should also visit a cardiologist to detect heart disorder or pulmonary hypertension secondary to pulmonary condition that might decompensate with altitude.

Patient education about risks associated with altitude and self-management seem to be a priority. Even if there is no study in this particular situation, it is a strategy that has proved efficient to decrease exacerbations and hospitalizations in COPD [69]. Recognizing complications and coping strategies, including medication use and inhaler technique, could help improve altitude tolerance.

Treatment optimization is also essential before traveling. Indeed, pharmacological therapies exist, allowing us to reduce symptoms or exacerbations, and to improve exercise tolerance. The classes of medications commonly used in COPD mainly include beta_2_-agonists and anticholinergics. They allow to improve expiratory flow and, thus, reduce dynamic hyperinflation at rest and during exercise [70,71]. It is essential to ensure if inhaler technique is correct, as major errors occur frequently and are associated with lower symptom control [72].

A recent study by Lichtblau, whose aim was to prevent right-to-left shunt by administration of dexamethasone, has not returned a reduction of the prevalence of this trouble at 3100 m [45]. By contrast, the same team provided interesting results about effects of dexamethasone on pulmonary hemodynamics in COPD patients going to altitude [73]. In this study, administration of dexamethasone 8 mg/d during a 3-day stay allowed a limit of the rise in trans-tricuspid pressure gradient. In the same population, this treatment compared to a placebo, improved nocturnal oxygen saturation by a mean of 3% (95%CI: 2–3%), decreased sleep apnea by a mean of 18.7 events/h (95%CI: 12.0–25.3), and improved subjective sleep quality [74].

Acetazolamide is a carbonic anhydrase inhibitor commonly used to prevent acute mountain sickness and to accelerate acclimatization [75]. Acetazolamide causes a metabolic acidosis by increasing retention of hydrogen ions and increasing renal excretion of bicarbonate, thus inducing an increase in respiratory drive [76]. Acetazolamide also causes a locally elevated PaCO_2_ in the brain and lower pH, further increasing ventilatory drive [77]. However, effects of carbonic anhydrase inhibitors are complex and may impact negatively patients who cannot raise their minute ventilation, such as sometimes in severe COPD [78]. Indeed, the lung also contains carbonic anhydrase, and inhibiting this activity could lead to impair ventilation-perfusion matching [79]. It could also inhibit hypoxic peripheral chemoreceptor responsiveness [80]. Last, carbonic anhydrase inhibitors reduce maximal inspiratory pressure due to muscle respiratory acidosis and, thus, likely decrease respiratory muscle performance [81]. Hence, this might lower PaO_2_ and increase PaCO_2_. Therefore, it should be used with caution in COPD patients.

In COPD patients suffering from dyspnea at sea level, pulmonary rehabilitation has considerable effects. It is defined by “a comprehensive intervention based on thorough patient assessment, followed by patient-tailored therapies that include, but are not limited to, exercise training, education, self-management, and interventions aiming at behavior changes, designed to improve the physical and psychological condition of people with chronic respiratory disease and to promote the long-term adherence to health-enhancing behaviors” [82]. It improves dyspnea, health status, and exercise tolerance [83]. Indeed, improvements in skeletal muscle function lead to improvements in exercise capacity. Moreover, the improved oxidative capacity of the skeletal muscles resulted in a decrease in ventilator requirement for a given work rate [82]. Such care could have considerable benefits for COPD patients wishing to travel in altitude, where maximal aerobic capacities are decreased.

As mentioned earlier, altitude increases the work of breathing. The diaphragm is responsible for about 60–70% of ventilation. In COPD patients, mechanical constraints are increased by bronchial resistances and decrease of parenchymal elasticity. Moreover, modification of conformation due to diaphragm flattening could result in a reduction of the diaphragm’s strength, even if there are probably coping mechanisms limiting this phenomenon [84,85,86]. In this context, inspiratory muscle training (IMT) could be of particular importance. In COPD patients at sea level, it confers gains in inspiratory muscle strength and endurance. There is also a reduction in dyspnea, even if not associated to whole-body exercise training [87,88]. In a preliminary investigation in 2010, IMT attenuated the fall in resting SpO_2_ in a population of 14 healthy people at the altitude of 4880 m and above. The SpO_2_ was 6% higher in the IMT group [89].

**Figure 2:**
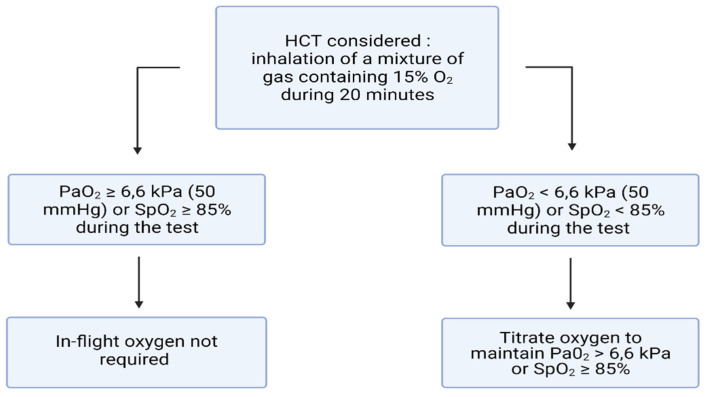
Conduct of hypoxic challenge testing in patients with pulmonary disease.

Oxygen supplementation is recommended if PaO_2_ reached 6.6 kPa, or SpO_2_ < 85% during HCT, by titrating oxygen during a second test while monitoring PaCO_2_ (to avoid oxygen-induced hypercapnia) (Figure 2). There is little evidence supporting the threshold of 6.6 kPa, but many physicians considered this cut-off reasonable [32,34]. To our knowledge, there is no study which has precisely evaluated this limit. Patients already with long-term oxygen treatment should double their oxygen flow [34].

In the case of air travel, patients should contact their airline companies to determine which oxygen delivery support could be authorized on board (portable oxygen concentrators or oxygen cylinders provided by the airline company as their own oxygen cylinder is prohibited for safety reasons). Indeed, regulations may differ between states. Moreover, travelers should check with oxygen supply companies to determine whether their specific portable oxygen concentrator is approved for in-flight use. There are some resources on line which can help people through these steps, provided by the European Federation of Allergy and Airways Diseases Patients’ Associations [90] or the Federal Aviation Administration (FAA). There is some advice for patients, such as arriving early at the airport to avoid rushing, warning the airport authority if any need of assistance is required, and carrying an appropriate supply of medication.

## 8. Conclusions

As altitude travels and air transports are increasing, more and more patients with respiratory illnesses, and especially COPD, are exposed to altitude. Every physician facing those people should know about altitude risks and ways to manage them. COPD patients in altitude are at high risk to develop hypoxemia, bronchospasm, dynamic distention, pulmonary hypertension, cardiac decompensation, and sleep disorders. Patients should undergo careful assessment, and caregivers should consider some specific interventions, such as treatment optimization, oxygen therapy, inspiratory muscle training, or pulmonary rehabilitation. Studies in this area are still scarce, and it would be of particular interest to develop them.

## Data Availability

Data sharing not applicable.
